# Association of skipping breakfast with depression: a systematic review and meta-analysis

**DOI:** 10.3389/fpsyt.2025.1548282

**Published:** 2025-08-05

**Authors:** Junwen Tan, Qingwei Meng, Cheng Luo, Shipeng Zhang, Enjie Tang, Yanjie Jiang, Sijing Cheng, Xueying Li, Ling Li

**Affiliations:** ^1^ Hospital of Chengdu University of Traditional Chinese Medicine, Chengdu, China; ^2^ Department of Neurology, 925 Hospital of People’s Liberation Army (PLA) Joint Logistics Support Force, Guiyang, Guizhou, China; ^3^ Nanjing Hospital of Chinese Medicine Affiliated to Nanjing University of Chinese Medicine, Nanjing, China; ^4^ Department of Nephrology, The Central Hospital of Dazhou City, Dazhou, Sichuan, China

**Keywords:** depression, skipping breakfast, meta-analysis, subgroup analysis, outcome analysis

## Abstract

**Objective:**

Depression is a significant global public health issue, and Breakfast habits may be related to its onset. This study conducted a meta-analysis of previous studies to analyze the correlation between breakfast and depression, comprehensively evaluated the association between skipping breakfast and the risk of depression, and explored the potential sources of heterogeneity.

**Methods:**

PubMed, Embase, and Web of Science databases were searched(the retrieval time limit for all was from the establishment of the databases to September 1, 2024), English documents were selected from the databases(the research type was observational study), and then the data was extracted and the Newcastle-Ottawa Scale(NOS) was evaluated for data analysis of the selected studies. This study followed the guidelines of the Preferred Reporting Project (PRISMA) and Prospero Registration Agreement. The mixed-effects model combines the maximum adjusted estimates and measures heterogeneity using the I^2^ statistic. Sensitivity analysis verified the robustness of the analysis and assessed publication bias.

**Results:**

A meta-analysis of 12 literatures showed that skipping breakfast was positively correlated with the incidence of depression (RR=1.83, [95%CI 1.52-2.20], τ^2^: 0.09, I^2^: 96.37%). Egger test was conducted on the relationship between skipping breakfast and depression, P=0.067 > 0.05, and the result suggested that there was no significant publication bias. Subgroup analysis indicates that current studies in different regions still have deficiencies, and the analysis shows that the occurrence of depression is associated with gender and the sample size of the study.

**Conclusions:**

Skipping breakfast can increase the risk of depression. It suggests that we should pay attention to having a regular and standardized breakfast to reduce the risk of depression. High heterogeneity may stem from differences in dietary culture and assessment methods. In the future, more research is needed to explore the mechanism and increase studies in different regions.

**Systematic Review Registration:**

https://www.crd.york.ac.uk/prospero/, identifier PROSPERO CRD42024583486.

## Introduction

Current surveys had found that mental illness is the leading cause of disability worldwide, with a lifetime prevalence of 12.2% to 48.6% in adults (1). The World Health Organization(WHO) reported that about 280 million people suffer from major depressive disorder, making it the leading cause of mental health-related disability worldwide (2). The survey showed that the prevalence of depression is gradually increasing, and it has now become a major public health problem, bringing huge economic and health costs to the government (3).

Depression is a major, widespread and global public health problem linked to many factors, such as diet, social factors and lifestyle habits. Eating behavior can have an impact on mental health through multiple pathways, including circadian rhythms, oxidative stress, and gut microbiota (4). In eating behavior, breakfast has a significant impact on body metabolism and circadian rhythms, and studies have shown that skipping breakfast is a growing trend that is significantly associated with an increased risk of overweight, obesity, cardiovascular disease, and type 2 diabetes (2). Breakfast is considered the most important meal (5), but the act of eating breakfast is often skipped in today’s society. In addition to increasing the risk of metabolic related diseases, skipping breakfast is also significantly associated with mental health such as depression, stress and anxiety (6), so eating behavior is considered to be an important factor in mental health (7).

Reviewed of previous studies on breakfast and depression, some studies found that eating breakfast every day can reduce the incidence of common mental disorders, mainly including depression and anxiety disorders (8). In today’s society, depression has become a global public health problem, any research that can reduce the risk of depression is of great significance. This study conducted an integrated analysis of the studies on skipping breakfast and depression, and mainly explored the correlation between skipping breakfast and depression, in order to provide data support for the risk of depression.

## Methods

### Study design

We conducted a systematic review and meta-analysis of studies on the association between skipping breakfast and depression. The entire process followed the Preferred Reporting Project for Systematic Review and Meta-Analysis (PRISMA) 2020 guidelines (9). The research program has been published in the International Systematic Review Prospective Registry (PROSPERO: Registration No.CRD42024583486).

### Search strategy

**Figure 1:**
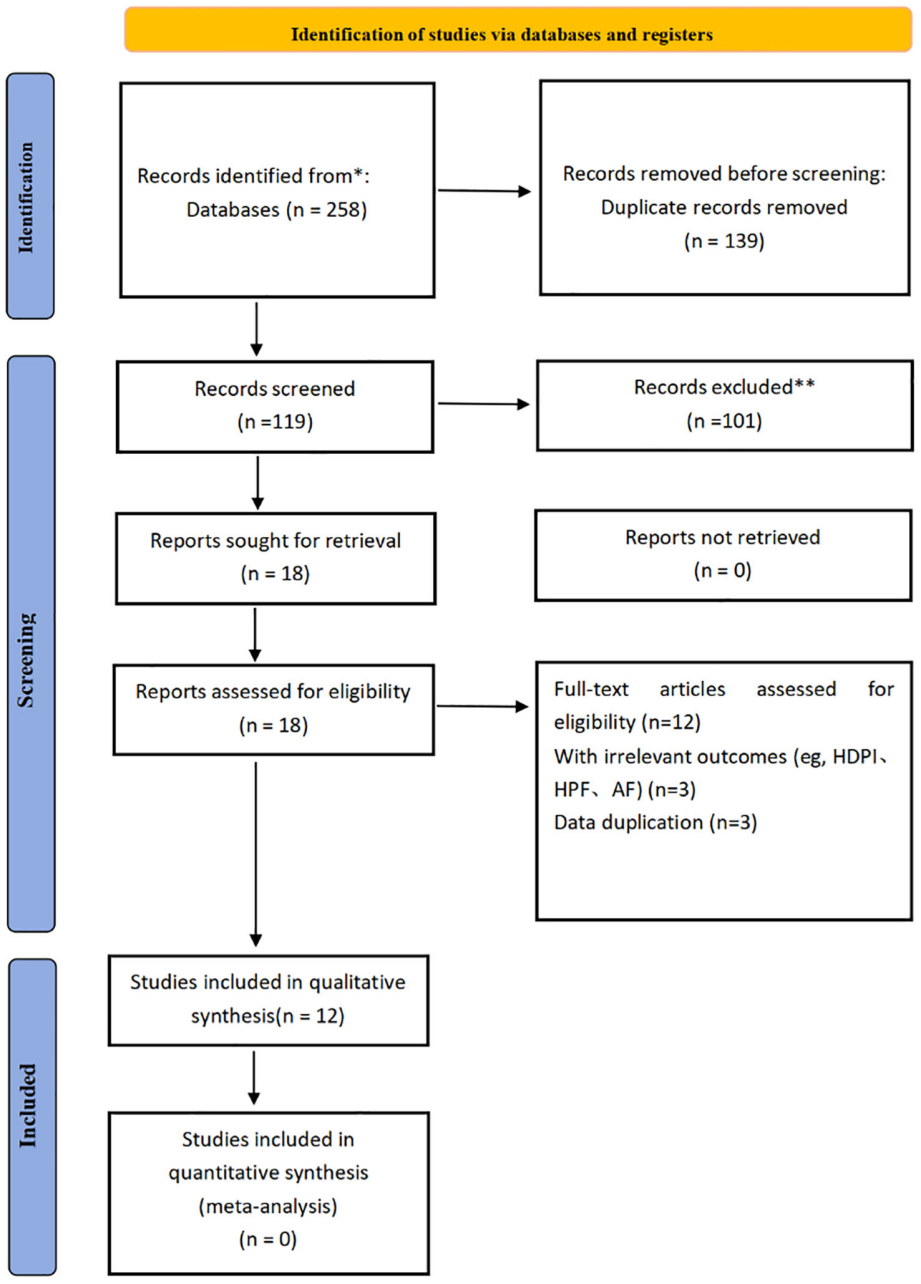
The literature screening process.

Case studies on the relationship between skipping breakfast and depression in PubMed, Web of Science, Embase and other databases were searched from the establishment of the database to September 1, 2024. The language of publication is limited to English. The search took the way of combining subject words and free words. English search terms include: “Meal Skipping”, “Skipping, Meal”, “Breakfast Skipping”, “Skipping, Breakfast”, “Depressive Disorder”, “Depression”, “Depressive Symptoms”, “Depressive Symptom”, “Symptom, Depressive”, “Emotional Depression”, “Depression, Emotional”, et al., manually searched a list of references for articles on similar topics to look for studies that might qualify. The detailed search process is shown in the following figure. The detailed search process and search strategy can be found in **Figure 1** and the appendix.

### Eligibility criteria

Inclusion criteria: (1) Subjects: The subjects were those who did not eat breakfast; In this study, according to the classification standard of skipping breakfast proposed by K Sharma (10), behaviors that eat breakfast no more than 2 times a week are regarded as skipping breakfast. (2) The incidence of depression in eating breakfast and skipping breakfast was concerned about the results; (3) The type of study is observational studies (cross-sectional, case-control, and cohort studies).

Exclusion criteria: (1) Duplicate literature or report the same cohort. (2) The data is incomplete and the corresponding OR/RR/RR cannot be calculated. (3) Research on the population of patients with relevant diseases. (4) non-relevant exposure outcomes (such as anxiety disorders, suicide, etc.). (5) non-relevant study designs (such as intervention studies, reviews, and meta-analyses).

### Assessment of plant-based dietary patterns

Different studies defined skipping breakfast differently, classifying subjects according to how often they ate breakfast, based on previous reports. In this study, the classification standard of skipping breakfast proposed by K Sharma was adopted (10), and the behavior of eating breakfast no more than 2 times a week was regarded as skipping breakfast.

### Study selection

The literature screening process was divided into two stages. In the initial phase, two researchers (TJW and ZSP) were responsible for collecting the relevant literature. All the literature collected was entered into EndNote X9 and duplicated items were automatically and manually removed. Eligible studies were identified by reviewing the title and abstract against the set inclusion and exclusion criteria. In the second stage, the studies in question are evaluated through a full-text review to determine which studies are suitable for inclusion in the meta-analysis. In the literature screening process, if two researchers have different opinions, a third researcher (LC) will be invited to participate in the discussion until a consensus is reached (see the attachment for detailed screening process).

### Data extraction and quality assessment

The first author used standardized data collection tables to extract relevant data from eligible studies, which the second author checked against the original article. The information extracted after verification includes: First author’s last name and year of publication, name of study (if applicable), geographical location, study duration, type of study design, demographic characteristics (e.g., mean age, proportion of women), sample size, number of cases exposed to the results and the circumstances under which they were determined (e.g., incidence of depression), method of assessment for depression, most fully adjusted risk ratio, adjusted in statistical models Confounding factors and risk of confounding diseases. Finally, the data extraction results are formed on the basis of the consensus reached by ZSP and LC. The quality of the studies was assessed using the NOS, which independently assessed the quality of the initially included studies. NOS consists of eight entries in three dimensions (selectivity, comparability, and outcome evaluation), setting a maximum score of 9 points for each study, with 4 points for selectivity evaluation, 2 points for comparability evaluation, and 3 points for outcome evaluation. Based on scores, studies were classified as low quality (0-3), medium quality (4-6), and high quality (7-9).Please refer to Appendix for the specific NOS rating scale.

### Data synthesis and analysis

To investigate the association between skipping breakfast and depression, we used Stata 16 to analyze the combined results of multivariate adjusted RR/RR/OR for each study. In the analysis, the HR ratio was treated as the RR, and the highest and lowest risk estimates were aggregated (11). Inter-study heterogeneity was measured using I^2^ statistics, and the results were visually demonstrated by forest maps. In I^2^ statistics, I^2^ ≤ 50% and I^2^>50% represent significant and insignificant heterogeneity, respectively. If the heterogeneity of results is obvious, the random effects model is used. On the contrary, the fixed effect model is used. To examine the significance of RR differences and the possible impact of residual confounders, a “leave-one-out” sensitivity analysis was performed by excluding one study per iteration to examine the contribution of individual studies to the overall effect. In addition, Egger test was used to evaluate the potential publication bias of the included studies and detect the impact of publication bias on the overall results.

## Results

**Table 1** showed the characteristics of eligible studies; A total of 12 literatures were included in this analysis (12–23). The literature included a total of 421,993 participants, with a sample size ranging from 108 to 207,710. 12 literatures on the association between skipping breakfast and depression. A systematic review of the association between skipping breakfast and depression related outcomes showed that skipping breakfast was associated with a higher incidence of depression. Some of the results showed significant heterogeneity, and subgroup analysis suggested that geographic region, sex ratio and total number of cases may be the sources of inter-study heterogeneity.

**Table 1:** The following are the basic characteristics of eligible studies.

Author	Data from	Study type	Time	Age	Female(%)	Case	Total	Assessment mode	Outcome	Adjust
Bao-Peng Liu 2022 (12)	the Centers for Disease Control and Prevention(CDC)	Observational study	2011-2019	/	49.90%	22969	73074	YRBSs	Depressive Symptoms	the variables age, sex, race, survey year, dietary behaviors—including vegetable, fruit, milk, and fizzy drink consumption—and breakfast consumption.as well as weight status.
Geoff P. Lovell 2015 (13)	Students enrolled at the University of the Sunshine Coast, Queensland	Observational study	2010	/	100.00%	192	588	DASS-21, PSQI	depression	adjusted for age, anxiety, and stress
Geoff P. Lovell 2015 (13)	Students enrolled at the University of the Sunshine Coast, Queensland	Observational study	2010	/	0%	55	163	DASS-21, PSQI	depression	adjusted for age, anxiety, and stress
Haibo Xu 2022 (14)	the Student Mental Health Center at a university in eastern China	Observational study	2022	20.96	42.70%	6051	12922	PHQ-9	depression	/
Luyao Zhang 2021	the annual health examination of adult employees in Harbin, China,	Observational study	2017	/	41.99%	620	1998	SDS	depression	age, sex, body mass index, physical activity, educational level, occupation, living status, smoking and drinking habits, hypertension, and diabetes.
Ryuji Furihata 2018 (16)	the Nihon University Sleep and Mental Health Epidemiology Project(NUSMEP)	Observational study	2009	/	52.50%	159	2334	CES-D	depressive symptoms	age group, sex, city size, educational achievement, marital status and other unhealthy lifestyle factors
Sang Ah Lee 2017 (17)	the Korean Centers for Disease Control and Prevention(CHS)	Observational study	2013	/	46.10%	11832	207710	The CHS question on the experience of depressive symptoms	depression	/
Sixuan Li 2024 (18)	in Ningbo, Zhejiang Province	Observational study	2022	/	47.82%	1815	11887	CES-D	depressive symptoms	age, sex, residence, school type, BMI, smoking, alcohol use, bully victimization, experiences of being beaten and scolded by parents, physical activity, and breakfast skipping (when sleep duration was the primary independent variable) or sleep duration (when breakfast skipping was the primary independent variable).
Subin PARK 2018 (19)	the 2016 Korea Youth Risk Behaviour Web-based Survey (KYRBS)	Observational study	2016	14.99	48.40%	/	65528	physical and mental health variables.	Depressive mood	sex, school grade, residential area, socioeconomic status, and other dietary behaviours
Supa Pengpid 2020 (20)	from 28 countries in Asia ,Africa and the Americas	Observational study	2013	20	58.40%	2834	21972	CES-D	Depression (severe)	age, sex, subjective economic status, residence status, country, social support, and religiosity.
Tingting Qiao 2023 (21)	quarantined college students in Shanghai China	Observational study	2022	25.67	69.30%	241	384	PHQ-9	depressive symptoms	age, sex (man/woman), quarantine duration, marital status (unmarried/married), education level (undergraduate/ postgraduate/ doctoral student), BMI, skipping breakfast(yes/no), regular physical exercise(yes/no), positive attitude towards COVID-19(yes/no), stomachache or abdominal pain(yes/no), nausea or dyspepsia(yes/no), and constipation or diarrhea(yes/no)
Yanjie Yu 2022	the Chongqing area	Observational study	2019	13.44	50.20%	2922	22373	CES-D	depressive	/
Zhongyu Ren 2020 (23)	the Chongqing Nursing Vocational College Physical Fitness and Health cohort study	Observational study	2018-2019	18.6	90.20%	108	1060	SDS	depressive symptoms	sex, age (continuous variable), BMI (≥30 kg/m2, ≥25 kg/m2 and <30 kg/m2 or not);only one child (yes or no), father education (senior high school or less, college or undergraduate), mother education (senior high school or less, college or undergraduate), parents’ marital status (married, widowed, divorced), smoking status (regularly, occasionally, never), drinking status (regularly, occasionally, never), PA (≥23 MET·h·week−1 or not), sleep duration (6–8 h or not), good sleep quality (yes or no).

YRBSs, The Youth Risk Behavior Surveys.

DASS-21, the Depression,Anxiety,and Stress Scale short form.

PSQI, the Pittsburgh Sleep Quality Index.

PHQ-9, T he patient health questionnaire 9-item depression scale.

SDS, the Self-Rating Depression Scale.

CES-D, the Center for Epidemiologic Studies Depression Scale.

**Figure 2:**
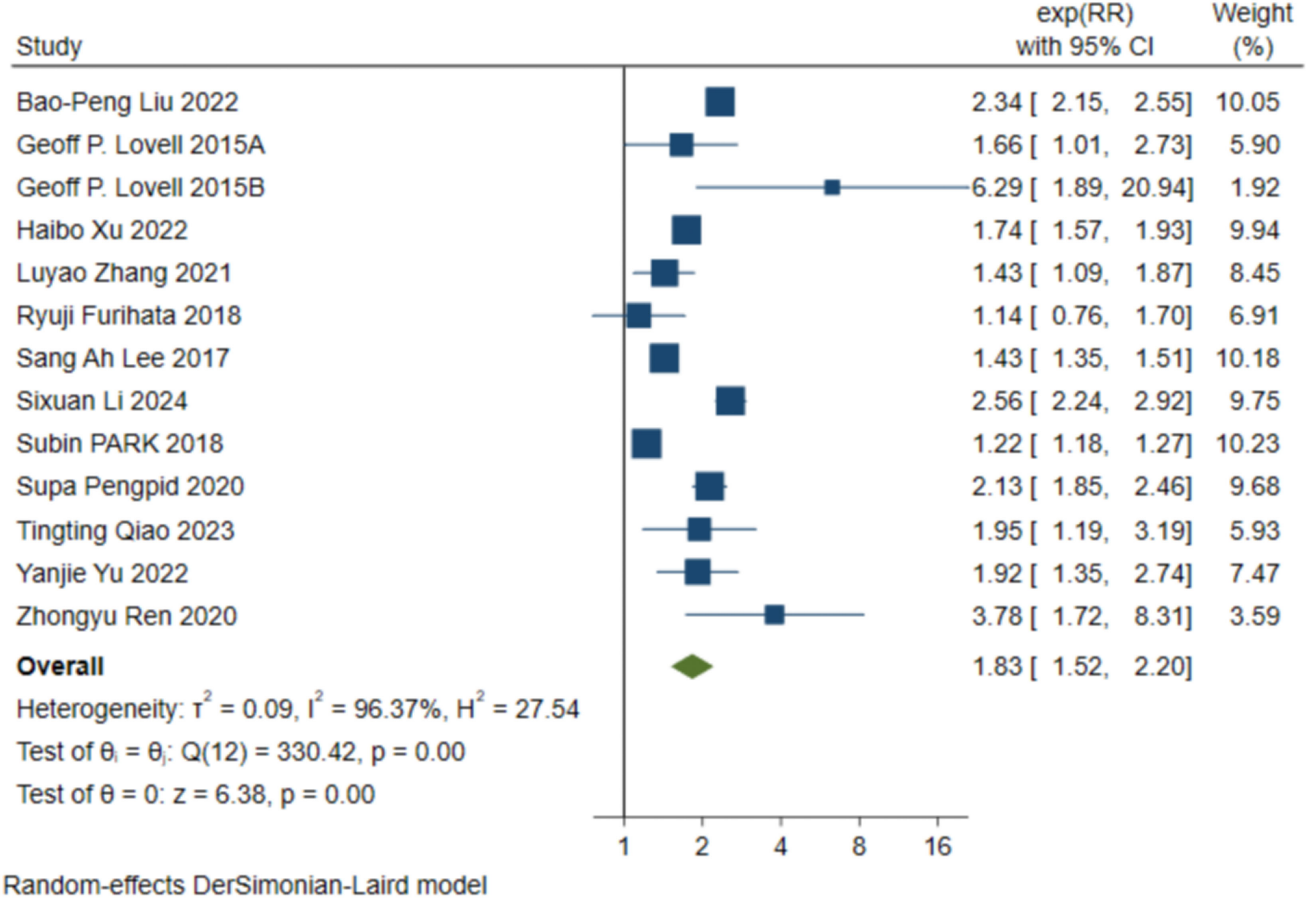
12 studies that included the relationship between skipping breakfast and depression were positively correlated (RR=1.83, [95% CI 1.52-2.20], τ 2: 0.09, 12:96.37%).

Twelve studies included an association between skipping breakfast and depression; The results showed that skipping breakfast increased the risk of depression (RR=1.83, [95%CI 1.52-2.20], τ^2^: 0.09, I^2^: 96.37%). Heterogeneity was high among studies. See **Figure 2** for a graph of the relevant data.

### Subgroup analysis

**Figure 3:**
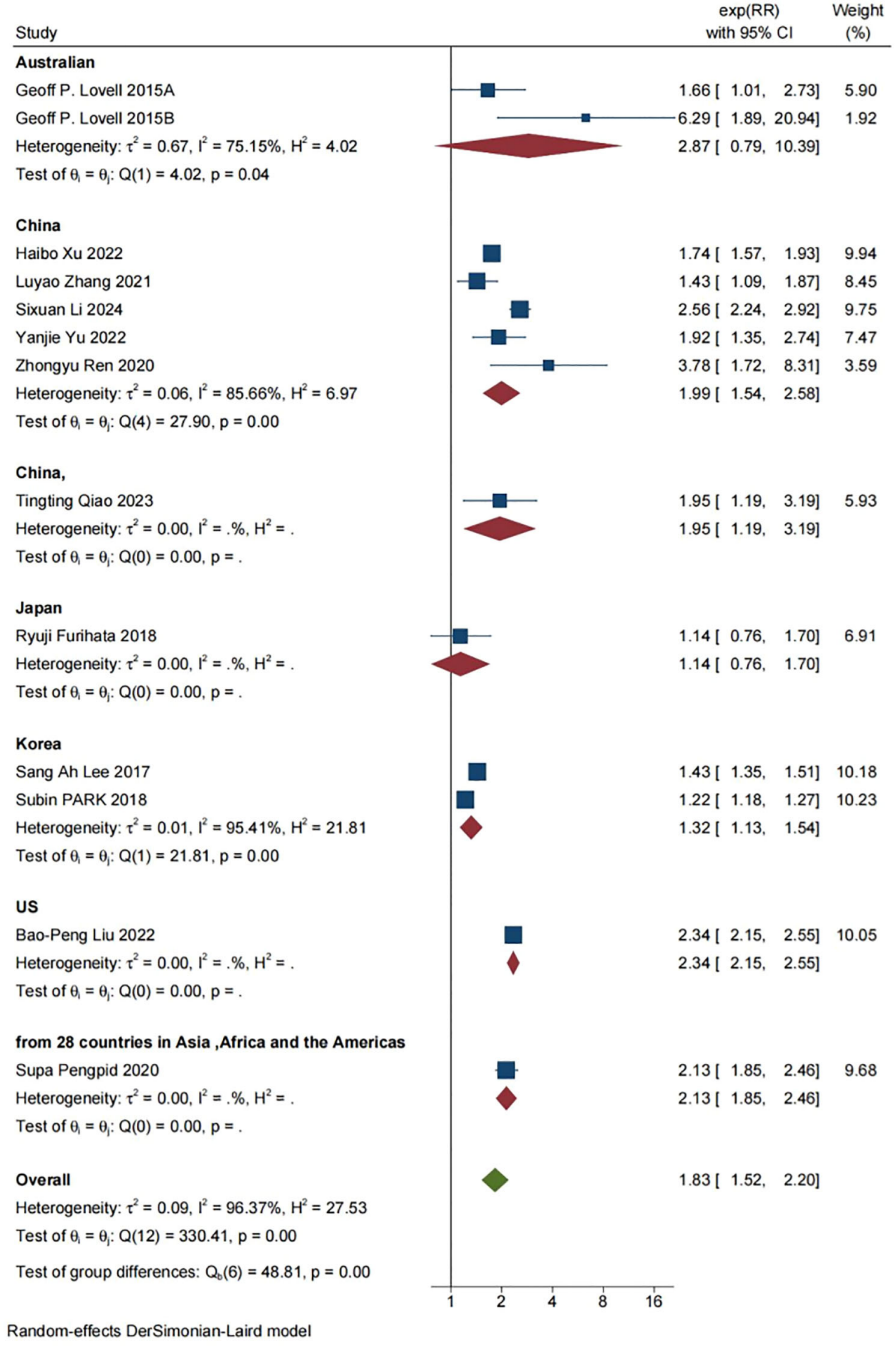
Subgroup analysis of the relevant regions of the selected literature (Concentrated in China, it showed a significant association with depression risk in all countries except Australia and Japan).

**Figure 4:**
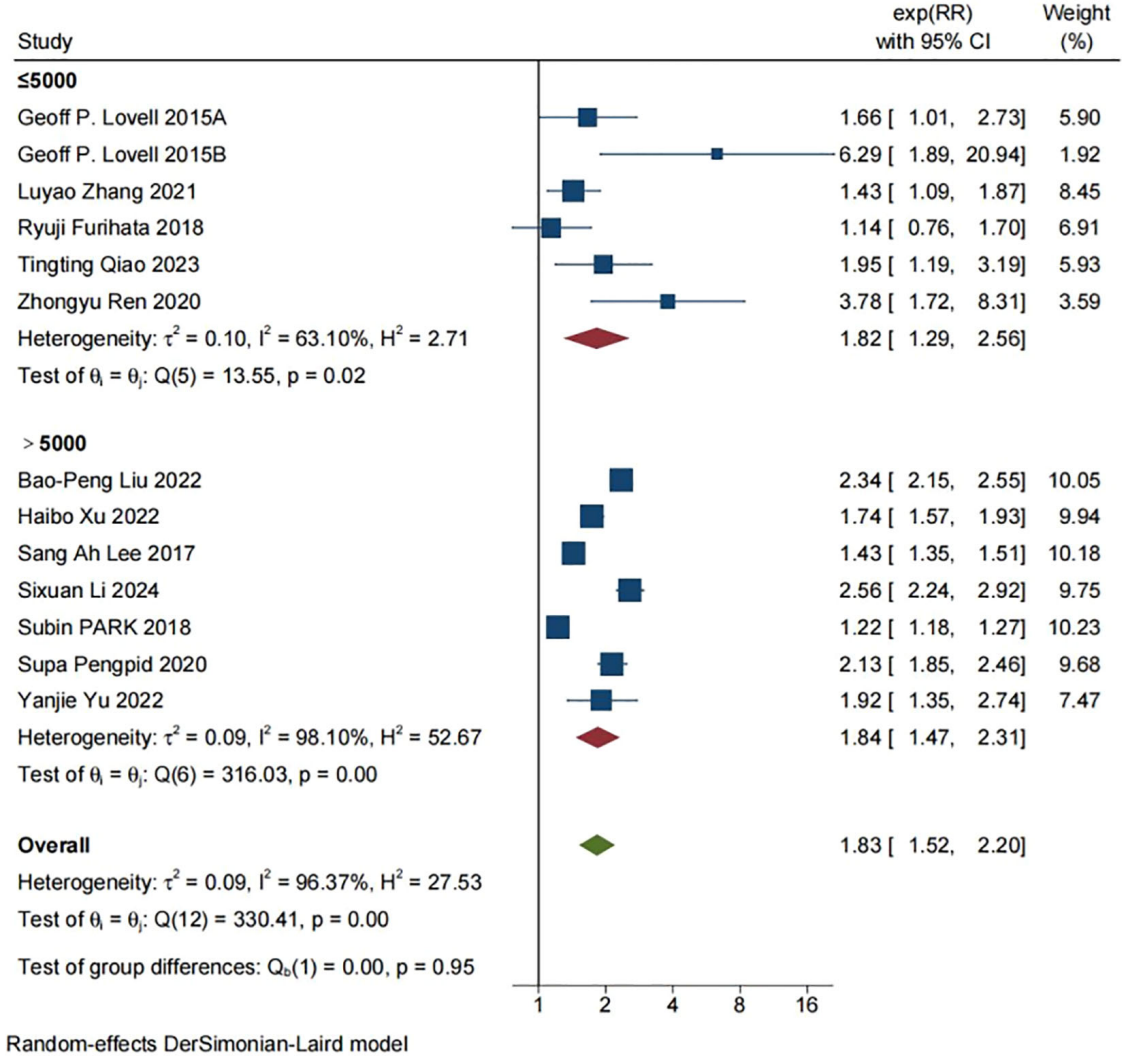
Subgroup analysis of the sample size of the selected literature (The results showed that the sample size was significantly associated with depression).

**Figure 5:**
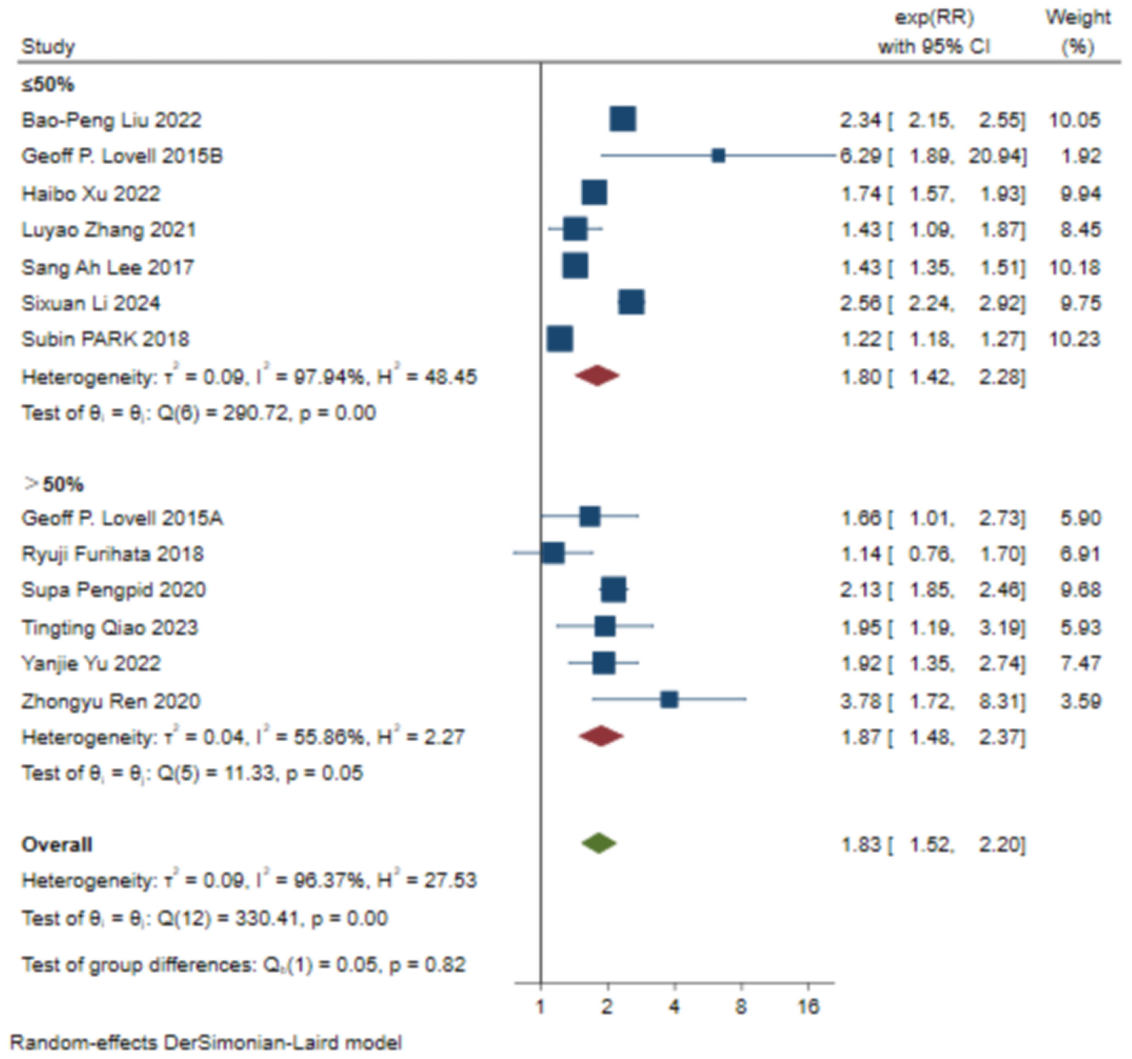
Subgroup analysis of the gender ratio of the selected literature (The results showed that the proportion of female was significantly associated with depression).

In order to further evaluate the correlation between covariate factors and results, we conducted stratified analysis of results based on region, proportion of female population, and total number of cases in each study. For detailed information, see **Table 2**, **Figures 3**, **4**, **5**.

**Table 2:** Subgroup analysis of the relationship between of breakfast and depression.

	No.of studies	RR (95% CI)	I^2^%	p for heterogeneity	Subgroup analysis data distribution map
**Location**					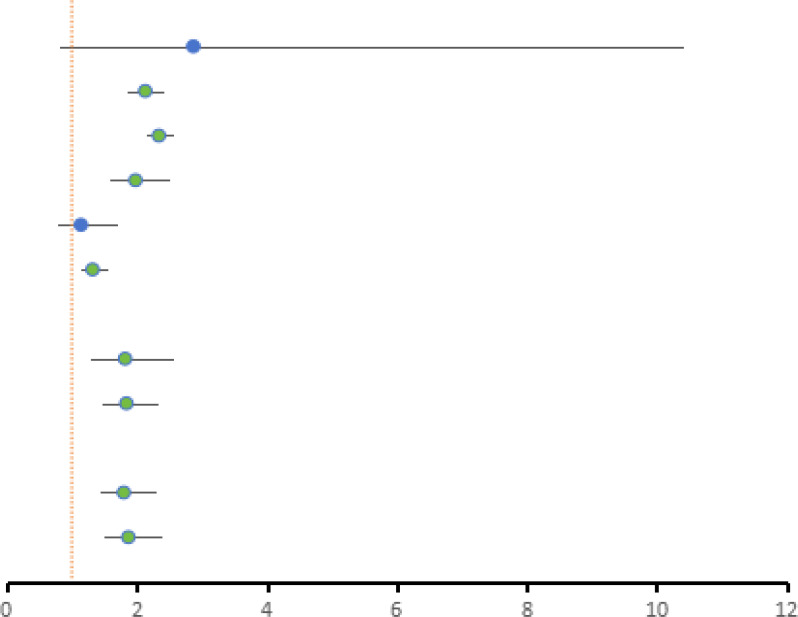
Australian	2	2.87(0.79,10.39)	0.75	0.04	
From 28 countries	1	2.13(1.85,2.46)	—	—	
US	1	2.34(2.15,2.55)	—	—	
China	6	1.98(1.57,2.50)	0.82	<0.01	
Japan	1	1.14(0.76,1.70)	—	—	
Korea	2	1.32(1.13,1.54)	0.95	<0.01	
**Total**					
≤5000	6	2.18(1.56,3.05)	0.76	<0.01	
>5000	4	1.82(1.38,2.40)	0.97	<0.01	
**Female**					
≤50%	7	1.80(1.42,2.28)	0.98	<0.01	
>50%	6	1.87(1.48,2.37)	0.56	0.05	
				

According to the data analysis of various studies on skipping breakfast, most of the current studies on the relationship between skipping breakfast and depression are concentrated in China, and further studies in different regions are needed to supplement them in the future. In addition, sample size may affect the accuracy of study results. In studies with sample size ≤5000, skipping breakfast was positively correlated with depression (RR=1.82, [95%CI 1.29-2.56], τ^2^: 0.10, I^2^: 63.10%). In the sample size >5000, there was a significant association between skipping breakfast and depression (RR=1.84, [95%CI 1.47-2.31], τ^2^: 0.09, I^2^: 98.10%). The comparison between the study with the sample size ≤5000 and the study with the sample size >5000 shows that the incidence of depression is more significant in the study with the sample size >5000, and the confidence interval is narrower and the outcome is more stable than that of the study with the sample size ≤5000. Among the effects of skipping breakfast on depression, sex ratio had a small effect, and skipping breakfast was positively associated with depression in studies with ≤50% female sample size (RR=1.80, [95%CI 1.42-2.28], τ^2^: 0.09, I^2^: 97.94%), and the association between skipping breakfast and depression was more significant in studies with > 50% women than in studies with ≤50% women (RR=1.87, [95%CI 1.48-2.37],τ^2^: 0.04, I^2^: 55.86%).

### Sensitivity analysis

**Figure 6:**
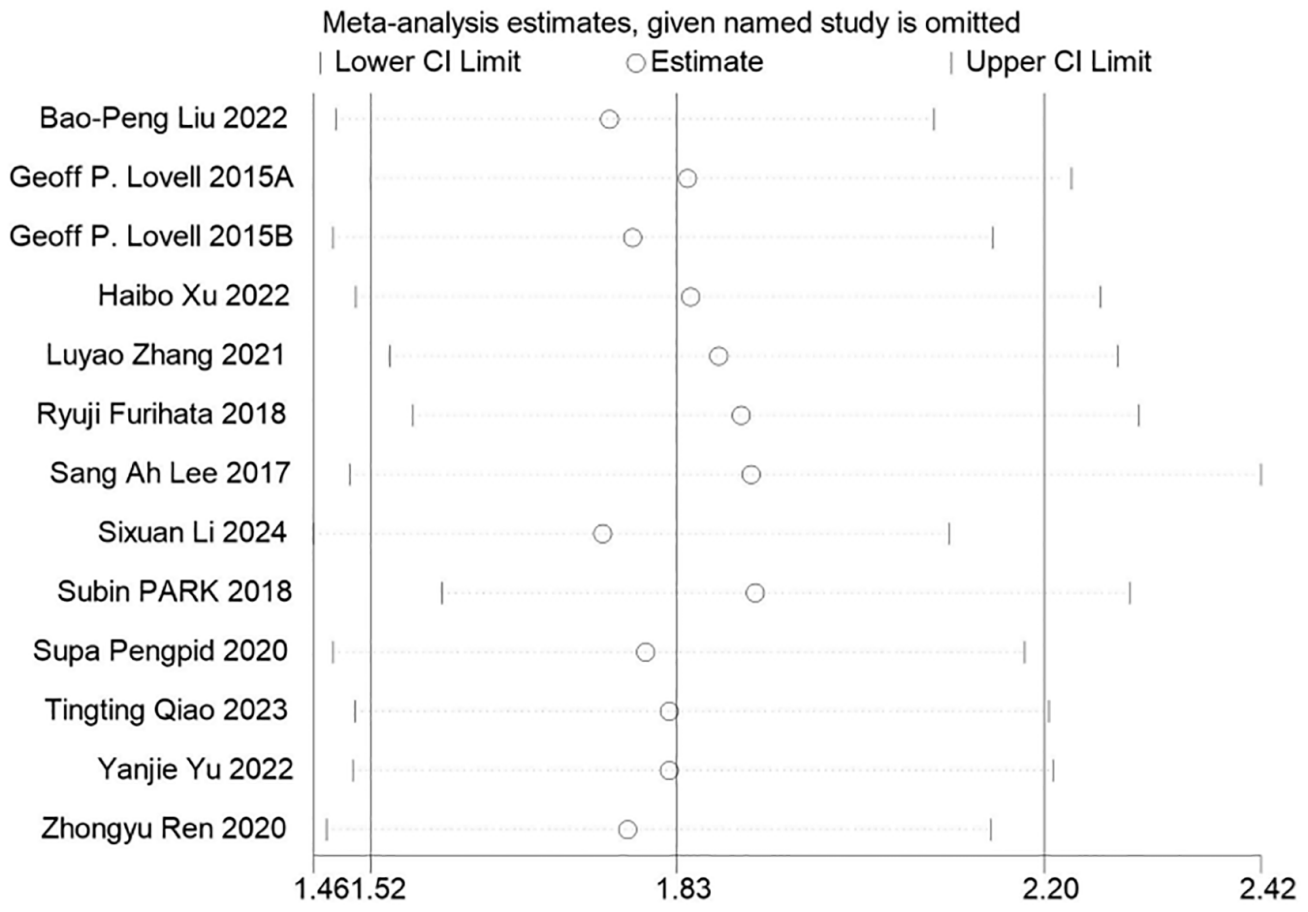
Sensitivity analysis was carried out on all the results, and the results were stable with no contradictory results.

Sensitivity analysis was carried out on all the results, and the results were stable with no contradictory results. See **Figure 6** for detailed analysis.

### Migration analysis

**Figure 7:**
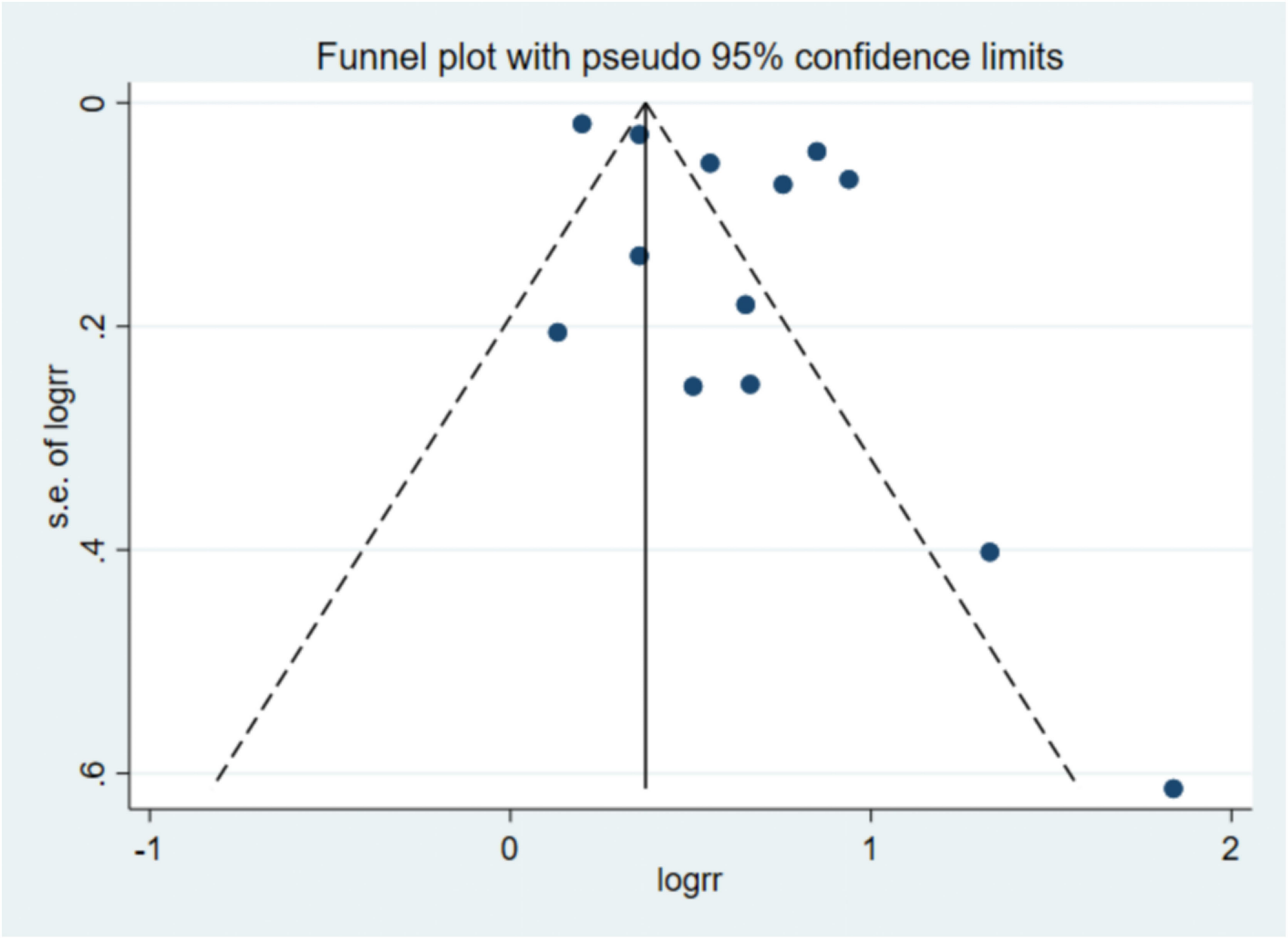
The deviation analysis results show that there is no obvious deviation (The results show that the data distribution is roughly symmetrical and the data is relatively concentrated, without obvious bias).

In this study, publication bias analysis was carried out on the included studies, and the analysis results showed that: In the analysis of the association between skipping breakfast and the risk of depression, **Figure 7** showed that the data distribution was roughly symmetric and the data were relatively concentrated, and the Egger test P=0.067 > 0.05 showed no significant publication bias.

## Discussion

Breakfast is one of the important behaviors for physical and mental health, and its importance lies in its multiple health benefits, including reducing obesity rate, cardiometabolic risk and improving quality of life (24, 25). In recent years, studies have also shown that skipping breakfast has been shown to be associated with type 2 diabetes (26), impaired cognitive function (27), and even cancer (28, 29).Psychologically speaking, breakfast is consumed after our longest post-meal fast. It provides the energy needed to start a new day after a night of fasting. At the same time, eating brings a sense of pleasure, which brings a good mood for work or study in the following day and has a positive and decisive impact on the quality of life and happiness (30, 31). Physiologically speaking, studies have shown that the disorder of biological clock gene expression is related to the asynchronous energy distribution of the biological clock (that is, low energy intake at breakfast), which can lead to circadian rhythm disorders and increase the risk of depression (32, 33). Meanwhile, circadian rhythm disorders can lead to melatonin regulation disorders, which in turn enhance sympathetic nerve tension, increase pulse index and norepinephrine levels, and increase the risk of cardiovascular disease (CVD) (34, 35). The chronic autonomic nervous system activation of depression, changes in the hypothalamic-pituitary-adrenal axis, metabolic disorders and immune inflammatory dysregulation are related to CVD (36). Meanwhile, studies have shown that it can reduce the level of cortisol in the human body, and depression caused by circadian rhythm disorders is related to elevated cortisol (37). Cortisol, as the main hormone of the hypothalamic-pituitary-adrenal axis, participates in the complex interaction of glucocorticoid release. Cortisol adjusts the neuron survival and neurogenesis. High levels of cortisol reduce neurogenesis, thereby leading to depression (38). In addition, the time to eat breakfast also has a certain impact on the human body. Currently, it is believed that the first meal eaten within 2 hours after the longest sleep in any 24 hours is breakfast (39), and the late meal time affects the eating of lunch, as well as metabolism and psychology.

The results of this analysis found that skipping breakfast was significantly associated with depression, although this association varied by gender and region, but all were positively correlated. Breakfast consumption is believed to have psychological effects, such as reducing anxiety, alleviating insomnia, and improving the quality of life. In the pathogenesis of depression, the imbalance of neurotransmitters, especially serotonin, dopamine and norepinephrine, plays a major role (40). Eating breakfast can have an impact on the gut microbiota (41), and the gut microbiota is related to various aspects of mental health. Previous studies have shown that gut microbiota can influence various physiological processes, including neurotransmitter production, immune responses and inflammation (42). On the one hand, the level of tryptophan in the systemic circulation is largely determined by the composition of the microbiota, which in turn indirectly determines the level of serotonin in the brain. On the other hand, some microbiota can regulate the synthesis of neurotransmitters (such as dopamine and norepinephrine), all of which are related to the occurrence and development of depression. The above reasons may together lead to a significant association between skipping breakfast and depression.

### Actual impact

In this study, we aimed to systematically analyze studies that assessed the association between skipping breakfast and depression. A total of 12 literatures on the association between skipping breakfast and depression were analyzed. From the overall analysis of 12 literatures, skipping breakfast is closely related to depression, and adhering to regular and nutritious breakfast will reduce the risk of depression.

### Advantage

This is a meta-analysis of skipping breakfast and depression. In this analysis, by adopting the standard definition of eating breakfast, most studies are summarized and defined, studies that do not meet the definition in different studies are excluded, studies that meet the definition are included, and the data offset caused by the screening literature is reduced through the integrated analysis of whether they meet the definition of eating breakfast. All the studies were included in the observational studies, and the literature quality was high, which ensured the reliability of the analysis results. The analysis results could effectively confirm the correlation between skipping breakfast and depression. For pooled analyses with high heterogeneity, subgroup analysis was used to identify some sources of study heterogeneity (region, proportion of female participants, total number of cases studied).

### Limitation

The research and analysis may still have certain limitations. First of all, the current meta-analysis involves sufficient sample size for the overall analysis, but the occurrence of diseases is closely related to regions and ages, and the study population in each region has different dietary customs. At the same time, due to the differences in work, wealth gap and living habits of people in each region, etc. Together, these factors lead to different attitudes toward breakfast in different regions, so that the results of studies on skipping breakfast and depression may be skewed. Second, there may be small study effects, which may threaten the validity of the meta-analysis results. We used a variety of methods to evaluate publication bias (that is, the main cause of the small study effect), including the asymmetry of the visual funnel plot and the Egger regression test (p=0.067 > 0.05), which showed that there was no bias in the study results, and the results showed that this extraction analysis was stable. However, due to the limited subgroup data available, more sources of heterogeneity cannot be identified in the current study. Finally, the occurrence of depression is not only related to whether or not you eat breakfast, but also to sleep, environment and other factors. Sleep quality can also affect the circadian rhythm of the subjects, leading to the occurrence of depression. In terms of environmental influences, both the family environment and external factors such as manganese can lead to the occurrence of depression. Some of the risk assessment models included in the study did not exclude relevant factors, which has a certain impact on the research results. In future studies on depression, the influence of multiple factors should be excluded and the influence of single factors should be emphasized. In order to provide more scientific data for the occurrence of depression.

## Conclusion

The results show that skipping breakfast has important physiological and psychological significance on the human body, which can increase the risk of depression. Therefore, it is very important to adhere to a regular and nutritious breakfast.

## Data Availability

The original contributions presented in the study are included in the article/**Supplementary Material**. Further inquiries can be directed to the corresponding authors.
