# Supplementary material for: Association of skipping breakfast with depression: a systematic review and meta-analysis
Source: Front Psychiatry. 2025 Aug 5;16:1548282. doi: 10.3389/fpsyt.2025.1548282 (PMC12362717; doi:10.3389/fpsyt.2025.1548282)
Supplement: Supplementary file 2 [file Supplementaryfile2.docx]

**eTable . NEWCASTLE - OTTAWA QUALITY ASSESSMENT SCALE**

| Author, year | Selection | | | | Comparability | Outcome | | | Overall  quality |
| --- | --- | --- | --- | --- | --- | --- | --- | --- | --- |
|  | Representative of cohort | Selection of cohort | Exposure ascertainment | No history of disease | Comparability of cohorts | Outcome assessment | Follow-up long enough(median ≥ 5 years) | Adequacy of follow up |  |
| Bao-Peng Liu 2022 | 1 | 1 | 0 | 1 | 1 | 1 | 0 | 0 | 5 |
| Geoff P. Lovell 2015 | 1 | 1 | 0 | 1 | 0 | 0 | 0 | 0 | 3 |
| Haibo Xu 2022 | 1 | 1 | 0 | 1 | 2 | 0 | 0 | 0 | 5 |
| Luyao Zhang 2021 | 1 | 1 | 0 | 1 | 2 | 0 | 0 | 0 | 5 |
| Ryuji Furihata 2018 | 1 | 1 | 0 | 1 | 2 | 1 | 0 | 0 | 5 |
| Sang Ah Lee 2017 | 1 | 1 | 0 | 1 | 0 | 1 | 0 | 0 | 4 |
| Sixuan Li 2024 | 1 | 1 | 0 | 1 | 2 | 1 | 0 | 0 | 5 |
| Subin PARK 2018 | 1 | 1 | 0 | 1 | 2 | 0 | 0 | 0 | 5 |
| Supa Pengpid 2020 | 1 | 1 | 0 | 1 | 2 | 0 | 0 | 0 | 5 |
| Tingting Qiao 2023 | 1 | 1 | 0 | 1 | 2 | 0 | 0 | 0 | 5 |
| Yanjie Yu 2022 | 1 | 1 | 0 | 1 | 0 | 0 | 0 | 0 | 3 |
| Zhongyu Ren 2020 | 1 | 1 | 0 | 1 | 2 | 0 | 0 | 0 | 5 |

**Supplemental Table** : Medical subject headings (MeSH) and non-MeSH terms used to search relevant publications on the relation between breakfast diets and depression ^1^

| Database | Step | Terms | Results |
| --- | --- | --- | --- |
| PubMed | 1 | ("Breakfast Skipping" [Mesh] OR "Skipping, Meal"[Mesh]) OR ("Breakfast Skipping"[Title/Abstract] OR "Skipping, Meal"[Title/Abstract] OR "Skipping, Breakfast"[Title/Abstract]) | 1037 |
|  | 2 | "Depression"[Mesh] OR "Depression"[Title/Abstract] OR "Depressive Disorder"[Title/Abstract] OR "Depressive Symptoms"[Title/Abstract] OR "Depressive Symptom"[Title/Abstract] OR "Emotional Depression"[Title/Abstract] | 532715 |
|  | 3 | #1 AND #2 | 71 |
| Web of Science | 1 | TS=("Breakfast Skipping" OR "Skipping, Meal" OR "Meal Skipping" OR "Skipping, Breakfast") | 1024 |
|  | 2 | TS=（"Depression" OR "Depressive Disorder"OR "Depressive Symptoms" OR "Depressive Symptom" OR "Symptom, Depressive"OR "Emotional Depression" ） | 411241 |
|  | 3 | #1 AND #2 | 86 |
| Embase | 1 | ('breakfast skipping'/exp OR 'skipping, meal'/exp) OR ('breakfast skipping':ti,ab,kw OR 'skipping, meal':ti,ab,kw OR 'skipping, breakfast':ti,ab,kw) | 1519 |
|  | 2 | 'depression'/exp OR 'depression':ti,ab,kw OR 'depressive disorder':ti,ab,kw OR 'depressive symptoms':ti,ab,kw OR 'depressive symptom':ti,ab,kw OR 'emotional depression':ti,ab,kw | 952776 |
|  | 3 | #1 AND #2 | 101 |

^1^ Two investigators searched the online databases independently.
